# Myocardial Energy Metabolism in Non-ischemic Cardiomyopathy

**DOI:** 10.3389/fphys.2020.570421

**Published:** 2020-09-16

**Authors:** Amanda A. Greenwell, Keshav Gopal, John R. Ussher

**Affiliations:** ^1^Faculty of Pharmacy and Pharmaceutical Sciences, University of Alberta, Edmonton, AB, Canada; ^2^Alberta Diabetes Institute, University of Alberta, Edmonton, AB, Canada; ^3^Mazankowski Alberta Heart Institute, University of Alberta, Edmonton, AB, Canada

**Keywords:** energy metabolism, cardiomyopathy, cardiac function, glucose oxidation, fatty acid oxidation

## Abstract

As the most metabolically demanding organ in the body, the heart must generate massive amounts of energy adenosine triphosphate (ATP) from the oxidation of fatty acids, carbohydrates and other fuels (e.g., amino acids, ketone bodies), in order to sustain constant contractile function. While the healthy mature heart acts omnivorously and is highly flexible in its ability to utilize the numerous fuel sources delivered to it through its coronary circulation, the heart’s ability to produce ATP from these fuel sources becomes perturbed in numerous cardiovascular disorders. This includes ischemic heart disease and myocardial infarction, as well as in various cardiomyopathies that often precede the development of overt heart failure. We herein will provide an overview of myocardial energy metabolism in the healthy heart, while describing the numerous perturbations that take place in various non-ischemic cardiomyopathies such as hypertrophic cardiomyopathy, diabetic cardiomyopathy, arrhythmogenic cardiomyopathy, and the cardiomyopathy associated with the rare genetic disease, Barth Syndrome. Based on preclinical evidence where optimizing myocardial energy metabolism has been shown to attenuate cardiac dysfunction, we will discuss the feasibility of myocardial energetics optimization as an approach to treat the cardiac pathology associated with these various non-ischemic cardiomyopathies.

## Introduction

Relative to its size, the heart possesses a metabolic demand that far exceeds that of any other organ in the body (Lopaschuk et al., 2010; Lopaschuk and Ussher, 2016). However, despite the extraordinary energy requirements necessary to sustain constant contractile function, basal metabolic processes, and ionic homeostasis, the heart has limited energy reserves and, therefore, must continually regenerate adenosine triphosphate (ATP) in order to maintain function. Although the heart relies preferentially on fatty acid metabolism to sustain a sufficient ATP supply, the heart acts omnivorously and demonstrates a unique capability to metabolize a variety of substrates in addition to fatty acids, such as carbohydrates (glucose and lactate), ketone bodies and amino acids (Lopaschuk et al., 2010; Lopaschuk and Ussher, 2016). This flexibility of substrate utilization allows the heart to accommodate alterations in substrate availability throughout various physiological states (e.g., nutrient ingestion, fasting). Of importance, this flexibility may deteriorate in response to pathological conditions where the heart must adjust its substrate preference to accommodate a perturbation in energy supply or demand, such as that seen during myocardial ischemia or heart failure.

With regards to the latter, heart failure is defined as a complex clinical syndrome that results from any structural or functional cardiac disorder that hinders the ability of the ventricle to fill with (diastolic dysfunction) or eject blood (systolic dysfunction) (Hunt et al., 2001). Despite the pathology of heart failure being multifactorial, it is generally accepted that the failing heart is energy-starved (Neubauer, 2007; Ussher et al., 2016). Myocardial ATP production is reduced by 30–40% in heart failure, therefore placing emphasis on the potential role of perturbed myocardial energetics in the mechanisms leading to heart failure. Chronic alterations in energy substrate utilization have been reported in heart failure, as well as in preceding cardiomyopathies (Neubauer, 2007; Taha and Lopaschuk, 2007; Lopaschuk et al., 2010). Furthermore, impaired metabolic flexibility has been postulated to contribute to the myocardial insulin resistance that characterizes the failing heart (Lopaschuk et al., 2010; Zhang et al., 2013). While heart failure may be the end diagnosis in an individual’s cardiac disease, many cases of heart failure are preceded initially by different forms of cardiomyopathy (Braunwald, 2017). Depending on the classification system used, cardiomyopathies can be classified as ischemic, dilated, hypertrophic, restrictive, inflammatory, arrhythmogenic, or diabetic to list a few. Given that alterations in myocardial energy metabolism are present in numerous cardiomyopathies that lead to heart failure, optimization of energetics during the cardiomyopathy stage may represent a promising therapeutic approach to attenuate or prevent subsequent heart failure development and progression.

Accordingly, we will review the primary metabolic pathways supporting cardiac function, the alterations in intermediary energy metabolism that accompany various cardiomyopathies, and whether these alterations can be targeted to mitigate cardiomyopathy and the eventual development of overt heart failure. We will not discuss energy metabolism in ischemic cardiomyopathy or dilated cardiomyopathy, as the perturbations of energy metabolism in these cardiac disorders have been extensively characterized in numerous reviews (Taha and Lopaschuk, 2007; Jaswal et al., 2011; Heusch et al., 2014; Gibb and Hill, 2018). Rather, we will focus our efforts on detailing the perturbations of intermediary energy metabolism accompanying various non-ischemic cardiomyopathies, including hypertrophic cardiomyopathy (HCM), diabetic cardiomyopathy, the cardiomyopathy associated with the rare genetic disorder, Barth Syndrome (BTHS), and arrhythmogenic cardiomyopathy.

## Energy Metabolism in the Healthy Heart

**FIGURE 1:**
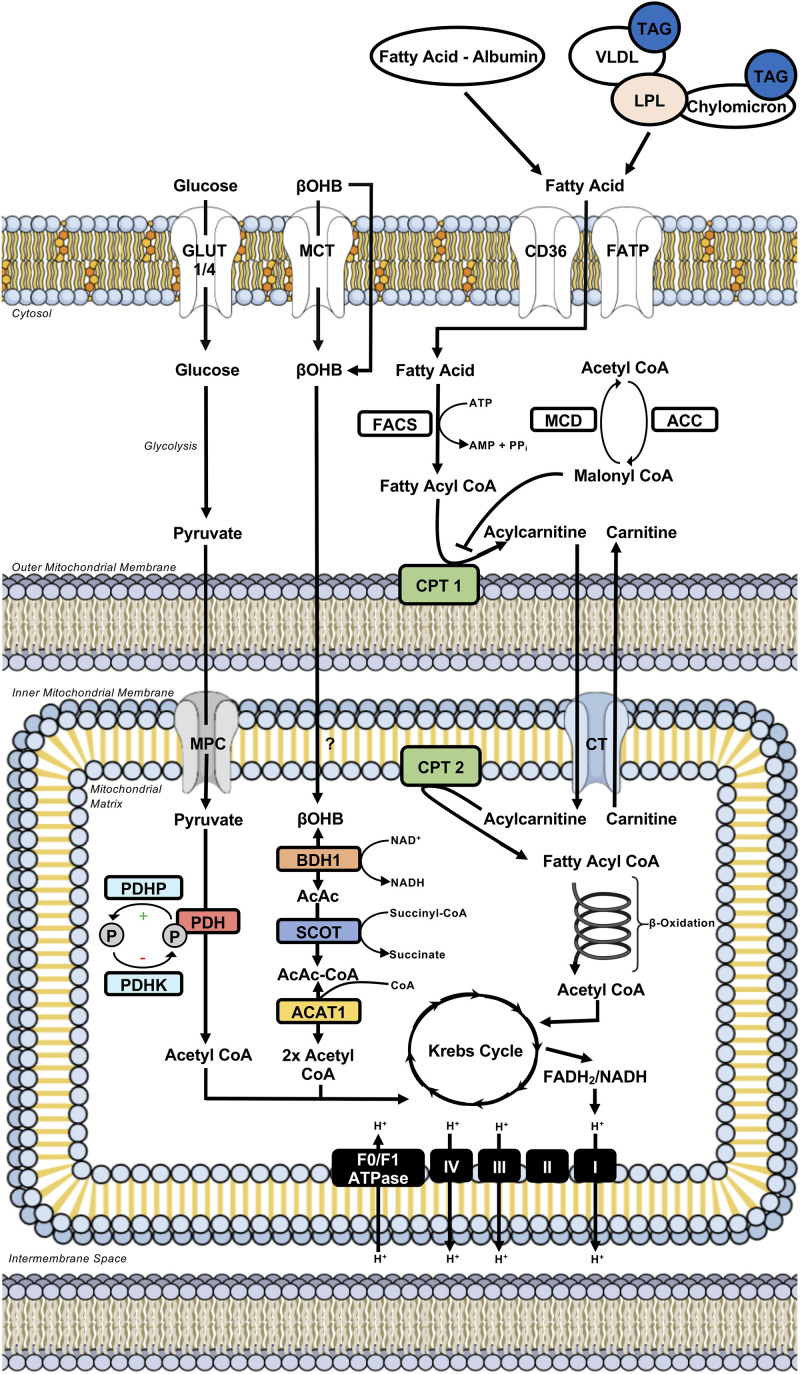
Intermediary energy metabolism in the cardiac myocyte. Illustration depicts intermediary metabolism of carbohydrates (glucose), fatty acids, and ketone bodies (βOHB) in the myocardium. Key nodes depicting the control of uptake into the cardiac myocyte, uptake into the mitochondria, and subsequent metabolism to acetyl CoA are highlighted. Acetyl CoA from the oxidation of carbohydrates, fatty acids, and ketone bodies is subsequently metabolized further in the Krebs Cycle. This results in the formation of reducing equivalents (e.g., FADH_2_/NADH) that donate their electrons to the complexes of the electron transport chain, driving oxidative phosphorylation and the ATP generation needed for sustaining contractile function. ACAT, acetoacetyl CoA thiolase; ACC, acetyl CoA carboxylase; BDH, β-hydroxybutyrate dehydrogenase; βOHB, β-hydroxybutyrate; CD36, cluster of differentiation 36; CPT, carnitine palmitoyl transferase; CT, carnitine translocase; FACS, fatty acyl CoA synthetase; FADH_2_, reduced flavin adenine dinucleotide; FATP, fatty acid transport protein; GLUT, glucose transporter; LPL, lipoprotein lipase; MCD, malonyl CoA decarboxylase; MCT, monocarboxylic acid transporter; MPC, mitochondrial pyruvate carrier; NADH, reduced nicotinamide adenine dinucleotide; PDH, pyruvate dehydrogenase; PDHK, PDH kinase; PDHP, PDH phosphatase; SCOT, succinyl CoA:3-ketoacid CoA transferase; TAG, triacylglycerol; VLDL, very-low density lipoprotein.

The heart derives the majority (∼95%) of its ATP production from the mitochondrial oxidation of fatty acids, carbohydrates (e.g., glucose, lactate), ketone bodies (e.g., acetoacetate, β-hydroxybutyrate), and amino acids, with the remainder being produced through aerobic glycolysis (Figure 1; Lopaschuk et al., 2010; Lopaschuk and Ussher, 2016). In the mature heart, fatty acids account for the majority of oxidative metabolism (50–70%), with the oxidation of glucose primarily accounting for the remainder. However, in response to nutrient ingestion, carbohydrates (glucose) can become the predominant fuel in the mature, metabolically flexible heart, due to the ensuing insulin response (Lopaschuk et al., 2010; Lopaschuk and Ussher, 2016). Although the heart is capable of metabolizing ketone bodies and amino acids, the majority of studies have demonstrated minimal contribution of these substrates to myocardial ATP production (Lopaschuk and Ussher, 2016). Conversely, recent studies in isolated working mouse hearts have demonstrated that ketone bodies can become a major fuel source for the heart, particularly at circulating concentrations representative of prolonged fasting/starvation (Ho et al., 2020), and thus we will also describe the regulation of myocardial ketone body oxidation herein. In addition to supporting ATP production, it is becoming more apparent that these substrates, or various intermediates arising from the metabolism of these substrates, can also influence cardiac function via directly regulating cellular signaling pathways. This can include post-translational modifications of proteins (e.g., acetylation, *O*-N-acetyl glucosaminylation), allosteric regulation of enzymes, or via transducing G-protein coupled receptors, though these aspects will be minimally discussed herein, as they have been extensively characterized in recent reviews (D’Souza et al., 2016; Lopaschuk and Ussher, 2016; Puchalska and Crawford, 2017; Murphy and O’Neill, 2018; Collins and Chatham, 2020; Ritchie and Abel, 2020).

### Fatty Acid Metabolism

The heart’s coronary circulation provides it with its fatty acid supply either as free fatty acids bound to albumin, or following the release of fatty acids from the hydrolysis of triacylglycerol (TAG)-containing lipoproteins or chylomicrons, mediated predominantly by the actions of lipoprotein lipase (Teusink et al., 2003; Lopaschuk et al., 2010). The majority of extracellular fatty acid uptake into the cardiac myocyte occurs through protein-mediated transport, which involves transporters such as cluster of differentiation 36 (CD36) and fatty acid transport proteins (FATP), whereas passive diffusion is thought to contribute to a smaller extent (Figure 1; Luiken et al., 1999; Kuang et al., 2004; Lopaschuk et al., 2010). Once present in the cytosol, fatty acids are rapidly activated for further metabolism via esterification to coenzyme A (acyl CoA) in an ATP-dependent manner by fatty acyl CoA synthetase.

Acyl CoAs have three primary metabolic fates in the heart, where they can be stored as TAG, utilized for the biosynthesis of membranes, and most importantly, due to the heart’s enormous energy demand, transported into the mitochondria for subsequent β-oxidation (Lopaschuk et al., 2010). Because the mitochondrial membrane is impermeable to long chain acyl CoAs, mitochondrial fatty acid uptake and subsequent β-oxidation is dependent upon a carnitine shuttle comprised of three enzymes. An outer mitochondrial membrane-localized carnitine palmitoyl transferase 1 (CPT-1) catalyzes the conversion of long chain acyl CoA into acylcarnitine for transport to the mitochondrial matrix by carnitine acyl translocase, following which the acyl CoA is regenerated via CPT-2 present in the inner leaflet of the inner mitochondrial membrane (Figure 1; Lopaschuk et al., 2010). Of importance, this carnitine shuttle regulating mitochondrial fatty acid uptake and subsequent β-oxidation is highly sensitive to regulation via malonyl CoA, a potent endogenous inhibitor of CPT-1. Malonyl CoA is the product of acetyl CoA carboxylation via acetyl CoA carboxylase, whereas it is degraded by malonyl CoA decarboxylase. Once inside the mitochondrial matrix, the CPT-2 regenerated acyl CoA is finally subjected to repeated cycles of β-oxidation, a process involving four enzymes (acyl CoA dehydrogenase, enoyl CoA hydratase, 3-hydroxyacyl CoA dehydrogenase, and 3-ketoacyl CoA thiolase) that progressively shortens the acyl CoA via 2 carbons released as acetyl CoA with each cycle (Figure 1). Acetyl CoA subsequently enters the Krebs Cycle, where it joins with oxaloacetate to form citrate and undergo a series of redox reactions. The Krebs Cycle results in the formation of reducing equivalents (reduced flavin adenine dinucleotide/nicotinamide adenine dinucleotide) that donate their electrons to the complexes of the electron transport chain (ETC) for the generation of ATP during oxidative phosphorylation (Figure 1). The heart also contains endogenous TAG stores that can be used to support ATP production, with some studies suggesting that fatty acids taken up into the heart are first shuttled through the intracellular TAG pool prior to undergoing mitochondrial β-oxidation (Shipp et al., 1964b; Banke et al., 2010).

### Glucose Metabolism

Myocardial glucose metabolism for energy production involves three major steps: glucose uptake, glycolysis, and the mitochondrial oxidation of glycolytically-derived pyruvate, a process referred to as glucose oxidation (Figure 1). Glucose uptake into cardiac myocytes is facilitated by glucose transporters (GLUT), of which GLUT4 and GLUT1 are responsible for insulin-dependent and insulin-independent glucose uptake, respectively (Brownsey et al., 1997; Jaswal et al., 2011). Glucose subsequently undergoes glycolysis in the cytosol, a metabolic pathway comprised of ten enzymes that result in the formation of minimal energy (2 ATP) and the three-carbon end-product, pyruvate. Glycolytically-derived pyruvate has two primary metabolic fates, where it can either be converted to lactate by lactate dehydrogenase or shuttled into the mitochondrial matrix by a monocarboxylic acid transporter [referred to as the mitochondrial pyruvate carrier (MPC)] for subsequent oxidation. In the healthy mature heart where oxygen is not limiting, the latter mechanism predominates and is regulated by the actions of pyruvate dehydrogenase (PDH), the rate-limiting enzyme of glucose oxidation (Patel and Korotchkina, 2006; Patel et al., 2014).

The PDH complex is a multienzyme complex that decarboxylates pyruvate into acetyl CoA, which has the same fate as fatty acid oxidation-derived acetyl CoA and enters the Krebs Cycle to generate reducing equivalents for supporting oxidative phosphorylation in the ETC (Figure 1). The PDH complex is intricately regulated by numerous post-translational modifications, including phosphorylation-mediated inactivation via four PDH kinase (PDHK) isoforms, and dephosphorylation-mediated activation via two PDH phosphatase isoforms (Figure 1; Patel and Korotchkina, 2006; Patel et al., 2014; Gopal et al., 2017). Recent studies have also demonstrated that PDH activity and subsequent glucose oxidation can be stimulated via sirtuin 3 mediated deacetylation (Jing et al., 2013). As many mitochondrial dehydrogenases including PDH are sensitive to calcium-mediated stimulation (Denton, 2009), it has been demonstrated that mitochondrial calcium uptake mediated by the mitochondrial calcium uniporter (MCU) can positively regulate PDH activity (Suarez et al., 2018).

The heart also contains endogenous glycogen stores from which glucose can be mobilized to support myocardial ATP production when needed. Another metabolic pathway of glucose includes the pentose phosphate pathway, which plays a major role in the production of reduced nicotinamide adenine dinucleotide phosphate needed to generate the endogenous antioxidant, reduced glutathione (Ussher et al., 2012). Although evidence suggests that perturbations in the pentose phosphate pathway may be implicated in the pathology of heart failure (Gupte et al., 2006), glucose flux through this pathway in the healthy mature heart is thought to be negligible (Shipp et al., 1964a; Ussher et al., 2012).

### Ketone Body Metabolism

β-hydroxybutyrate and acetoacetate are the two primary ketone bodies that the heart utilizes to support energy production. While both are able to enter the cardiac myocyte via passive diffusion, at elevated concentrations it has also been suggested that ketone body uptake may involve monocarboxylic acid transporters (Halestrap, 1978; Puchalska and Crawford, 2017). β-hydroxybutyrate is converted into acetoacetate via the actions of β-hydroxybutyrate dehydrogenase (BDH1). Acetoacetate is subsequently activated for metabolism via esterification to CoA as acetoacetyl CoA, which is catalyzed via succinyl CoA:3-ketoacid CoA transferase (SCOT). Acetoacetyl CoA thiolase then hydrolyzes acetoacetyl CoA into two molecules of acetyl CoA, which are subject to the same metabolic fate as either glucose or fatty acid oxidation-derived acetyl CoA, thereby entering the Krebs Cycle to generate reducing equivalents for supporting oxidative phosphorylation (Figure 1).

### Substrate Competition for Oxidative Metabolism

A significant contributor to the heart’s robust metabolic flexibility to switch between fatty acids and glucose as major fuel sources during fasting and nutrient ingestion, respectively, involves substrate competition for oxidative metabolism. Joseph Shipp and colleagues were the first to demonstrate that increasing fatty acid availability to the heart leads to a marked inhibition of glucose oxidation (Shipp et al., 1961). However, credit for the reciprocal relationship by which fatty acids and glucose compete for oxidative metabolism (glucose/fatty acid cycle) is attributed to the work of Randle et al. (1963), and is thus often referred to as the “Randle Cycle.” A plethora of both animal and human studies have provided strong evidence to support substrate competition between fatty acids and glucose for oxidative metabolism in the heart (Wisneski et al., 1985; Wisneski et al., 1990; Liu et al., 2002; Buchanan et al., 2005). Conversely, recent evidence has suggested that increasing the heart’s reliance on ketone bodies as an oxidative fuel source is not subject to the same reciprocal relationships that would be conjectured to lead to reduced glucose and fatty acid oxidation rates (Ho et al., 2020). Of relevance to the remainder of this review, numerous cardiac disorders including various cardiomyopathies and/or heart failure, are accompanied by metabolic alterations whereby this metabolic flexibility becomes impaired, which may directly contribute to their associated cardiac pathologies.

## Energy Metabolism in Hypertrophic Cardiomyopathy

HCM is characterized by a thickening of the left or right ventricular wall secondary to cardiac myocyte hypertrophy and in the absence of ventricular dilation. With an estimated prevalence of 1:200, HCM is reported as the most frequent form of inherited cardiomyopathy (Semsarian et al., 2015; Braunwald, 2017; Marian and Braunwald, 2017). Our knowledge of the pathology of HCM has greatly improved through major advancements in hemodynamics and genetics, with pivotal studies by Christine and Jonathan Seidman uncovering a key role for a missense mutation in the β-myosin heavy chain gene (*MYH7*) causing familial HCM in a French Canadian family (Geisterfer-Lowrance et al., 1990). Presently, more than 1400 mutations have now been identified as being associated with a HCM phenotype, mainly in genes encoding for sarcomeric proteins (Ho et al., 2015). Numerous studies have proposed that mutation-induced changes in sarcomere function may be attributed to inefficient sarcomere contraction leading to myocardial ATP depletion in HCM (Chandra et al., 2005; Witjas-Paalberends et al., 2014). Furthermore, imaging studies have revealed that inefficient cardiac contractility may precede the development of HCM, as demonstrated by reduced myocardial external efficiency in asymptomatic mutation carriers (Timmer et al., 2011; Guclu et al., 2017). Therefore, myocardial energetics may be disturbed and potentially contribute to cardiac dysfunction in genetic/inherited HCM. However, the influence of HCM-associated mutations on myocardial energy metabolism requires further investigation as measurements of myocardial metabolic flux in humans with genetic causes of HCM is limited. On the contrary, a considerable number of studies have investigated the role of altered myocardial energy metabolism during the progression of pathological cardiac hypertrophy, and whether optimizing myocardial energy metabolism may be beneficial. Although there is much ongoing debate regarding the energy metabolism profiles that characterize the hypertrophied heart (summarized in Table 1), these studies may provide insight into the potential metabolic disturbances that contribute to cardiac dysfunction (primarily diastolic dysfunction) in genetic/inherited HCM.

**TABLE 1:** Alterations in myocardial energy metabolism in cardiac hypertrophy.

	Experimental model	Alteration in myocardial metabolism	References
Fatty acid metabolism	Human failing heart samples and spontaneously hypertensive heart failure-prone rats	mRNA expression of fatty acid oxidation enzymes↓	Sack et al., 1996
	Human fetal, non-failing and failing adult heart samples	mRNA expression of fatty acid oxidation enzymes↓	Razeghi et al., 2001
	Male Wistar-Kyoto rats with AAC	Fatty acid oxidation↓	Allard et al., 1994
	Male C57BL/6J mice with AAC	Fatty acid oxidation↓	Zhang et al., 2013
	Male dogs with pacing-induced decompensated heart failure	Fatty acid oxidation↓	Osorio et al., 2002
	Male C57BL/6J mice with TAC	Fatty acid oxidation↓	Byrne et al., 2016
Glucose metabolism	Male Wistar-Kyoto rats with AAC	Glycolysis↑	Allard et al., 1994
	Male C57BL/6J mice with AAC	Glycolysis↓ Glucose oxidation↓	Zhang et al., 2013
	Dogs with pacing-induced decompensated heart failure	Glucose oxidation↑	Osorio et al., 2002
	Male C57BL/6J mice with TAC	Glucose oxidation↓	Byrne et al., 2016
	Male Wistar rats with AAC	Glucose uptake↑ Glycolysis↑	Tian et al., 2001; Nascimben et al., 2004
	AMPK γ2 subunit R302Q transgenic mice	Glucose uptake↑ Glycogen content↑	Folmes et al., 2009
Ketone body metabolism	Male and female C57BL/6J mice with TAC	Ketone body oxidation↑	Aubert et al., 2016; Ho et al., 2019

A striking hallmark of pathological cardiac hypertrophy and subsequent heart failure is the reversion to fetal gene expression patterns, which are associated with reductions in fatty acid oxidation and a concurrent increase in glucose utilization (Sack et al., 1996; Razeghi et al., 2001; Stanley et al., 2005). Reductions in protein expression and activity of fatty acid oxidation enzymes are only observed during the decompensated heart failure stage in spontaneously hypertensive and heart failure-prone rats, though mRNA expression is already markedly reduced in these rats at the compensated hypertrophy stage (Sack et al., 1996). Moreover, in male Wistar-Kyoto rats subjected to abdominal aortic constriction (AAC) for an 8 week duration to induce compensated hypertrophy, palmitate oxidation rates are reduced during aerobic perfusion of isolated working hearts compared to sham-operated rats (Allard et al., 1994). Similarly, AAC in male C57BL/6J mice also results in a reduction in fatty acid oxidation rates during aerobic isolated working heart perfusions (Zhang et al., 2013). This result is observed at 2 weeks post-surgery in C57BL/6J mice, a time-point where normal systolic function [e.g., left ventricular ejection fraction (LVEF)] but abnormal diastolic function (e.g., reduced e′/a′ ratios) are observed, reminiscent of cardiac function profiles often seen in cases of genetic HCM (Braunwald, 2017; McKenna et al., 2017). These observations in *ex vivo* models are also observed *in vitro*, as palmitate oxidation rates are markedly reduced in neonatal rat cardiac myocytes subjected to experimental hypertrophy via treatment with the α_1_-adrenergic receptor agonist, phenylephrine (100 μM) (Barger et al., 2000).

In cardiac hypertrophy, the consensus regarding elevations in glucose utilization is defined by an enhancement of glucose uptake and glycolysis, with inconsistent changes in glucose oxidation (Jaswal et al., 2011), further contributing to an altered metabolic profile recapitulating that seen in the fetal heart (Huss and Kelly, 2005). Indeed, aerobic isolated working heart perfusions performed in male Wistar-Kyoto rats subjected to AAC for an 8 week duration demonstrate significant increases in glycolysis rates with no change in glucose oxidation rates (Allard et al., 1994). Unlike the similarities previously discussed for fatty acid oxidation, studies in C57BL/6J mice subjected to AAC do not support these metabolic perturbations regarding glucose utilization (Zhang et al., 2013). Glycolysis and glucose oxidation rates were both diminished during aerobic isolated working heart perfusions in the presence of insulin concentrations (100 μU/mL) matching those used in the study in male Wistar Kyoto rats, whereas glycolysis and glucose oxidation rates were similar in the absence of insulin.

Reasons for the discrepancies in the energy metabolism profiles identified in the rat (Allard et al., 1994) and mouse (Zhang et al., 2013) studies just described remain unclear, however, they may involve species-specific differences, differences in perfusate conditions, or duration of the aortic constriction. Furthermore, while AAC in the male Wistar-Kyoto rats did produce significant cardiac hypertrophy, assessments of *in vivo* cardiac function were not performed, and thus it cannot be discerned whether both systolic and diastolic dysfunction was present in these rats. Conversely, serial assessments via ultrasound echocardiography were performed in the male C57BL/6J mice subjected to AAC. At the 2 week post-surgery time point when myocardial energy metabolism profiles were measured, these mice exhibited signs of diastolic dysfunction (reduced e′/a′ ratios), without systolic dysfunction (normal LVEF), consistent with cardiac function profiles observed in HCM. The degree of dysfunction is, therefore, an important aspect to consider, as the presence of systolic dysfunction contributes to a decompensated heart failure phenotype where general reductions in overall oxidative metabolism, and increases in glycolytic rates to counteract these reductions, are more likely to be observed (Osorio et al., 2002; Byrne et al., 2016).

Alterations in the transcriptional regulation of genes involved in mitochondrial oxidative metabolism may contribute to the decline in fatty acid oxidation observed in pathological cardiac hypertrophy. Myocardial mRNA expression of peroxisome proliferator-activated receptor-α (PPARα), a master regulator of fatty acid metabolism, is reduced in male Sprague-Dawley rats and C57BL/6 mice following AAC and transverse aortic constriction (TAC), respectively (Barger et al., 2000; Akki et al., 2008). This leads to reductions in the expression of numerous PPARα downstream target genes, including CPT-1 and medium chain acyl-CoA dehydrogenase. Cardiac PPARγ activity has also been linked to cardiac hypertrophy, as cardiac-specific PPARγ deficient mice exhibit a robust increase in LV size/mass (Duan et al., 2005). Conversely, cardiac-specific PPARγ overexpressing mice also demonstrate a robust cardiac hypertrophy, which is actually associated with elevations in mRNA expression of genes regulating fatty acid oxidation (e.g., CPT-1, acyl CoA oxidase) (Son et al., 2007). Reasons for these discrepancies are currently unknown, and surprisingly, deletion of PPARα alleviates the cardiac dysfunction present in cardiac-specific PPARγ overexpressing mice without impacting cardiac hypertrophy, yet actually increases myocardial fatty acid oxidation (Son et al., 2010). Reductions in myocardial carnitine content have also been observed in pathological cardiac hypertrophy in male Sprague-Dawley rats following AAC, which could contribute to decreases in fatty acid oxidation (Reibel et al., 1983).

Cardiac hypertrophy-related changes in glucose metabolism are more difficult to explain, due in part to inconsistency in the reported alterations, especially with regards to glucose oxidation. Nonetheless, despite reported differences on whether cardiac hypertrophy leads to increases, decreases, or no change in myocardial glucose oxidation rates, increases in glycolysis rates are often reported and significantly higher than glucose oxidation rates. This results in a marked uncoupling of glucose oxidation from glycolysis, thereby leading to increases in proton production and subsequent contractile inefficiency, as ATP is diverted away from supporting contractile function toward relieving intracellular acidosis (Liu et al., 2002; Jaswal et al., 2011). It has been postulated that the replenishment of Krebs Cycle intermediates via increases in anaplerosis may contribute to this uncoupling of glucose metabolism in cardiac hypertrophy (Des Rosiers et al., 2011). In male Sprague Dawley rats subjected to TAC-induced pressure overload, increases in myocardial anaplerosis are attributed to elevations of glycolytic pyruvate entering the mitochondria via malic enzyme mediated carboxylation to malate (Sorokina et al., 2007). Interestingly, cardiac hypertrophy related increases in glycolysis do not correlate with increased expression of glycolysis enzymes, however, may be due to increases in 5′AMP activated protein kinase (AMPK) activity (Tian et al., 2001; Nascimben et al., 2004), a cellular energy sensor that promotes ATP production during low energy states (Steinberg and Kemp, 2009). Indeed, increases in myocardial AMPK activity have been observed in male Wistar rats at least 17 weeks after ascending aortic constriction, resulting in increased GLUT1 protein expression and subsequent 2-deoxyglucose uptake (Tian et al., 2001). In addition, the elevation in myocardial AMPK activity augments phosphofructokinase-2 mediated formation of fructose-2,6-bisphosphate, a potent allosteric stimulator of phosphofructokinase-1, the rate-limiting enzyme of glycolysis (Nascimben et al., 2004). It should be noted, though, that since *in vivo* cardiac function was not monitored in these rats, it is not possible to discern whether these metabolic alterations are due to potential systolic dysfunction rather than cardiac hypertrophy *per se*. Nonetheless, alterations in AMPK activity are a reasonable molecular culprit with regards to HCM, as mutations in the AMPK γ2 subunit have been shown to induce familial HCM in humans (Gollob et al., 2001; Taha and Lopaschuk, 2007; McKenna et al., 2017). One notable mutation involves a missense mutation resulting in an arginine substitution for glutamine at position 302 of the AMPK γ2 subunit (R302Q), which causes a glycogen storage HCM characterized by a familial form of Wolff-Parkinson-White syndrome. Key studies by Folmes et al. (2009) demonstrated that adenoviral-mediated overexpression of an AMPK γ2 subunit R302Q mutant increases AMPK activity, glucose uptake, glycogen synthase protein expression, and glycogen content in neonatal rat ventricular cardiac myocytes. Moreover, transgenic mice harboring a cardiac myocyte-specific AMPK γ2 subunit R302Q mutation also exhibit robust cardiac hypertrophy that is associated with massive glycogen accumulation (Folmes et al., 2009). While the described studies support a key role for increased AMPK activity and its metabolic actions in the pathology of HCM, AMPK also has contrasting anti-hypertrophic actions that need to be considered, such as inhibiting protein synthesis (Chan and Dyck, 2005).

Of interest, it has now been demonstrated in preclinical and clinical studies that ketone body oxidation is increased in both compensated hypertrophy and heart failure (Lopaschuk and Ussher, 2016; Ussher et al., 2016; Ho et al., 2019). Compensated hypertrophy in female C57BL/6J mice in response to TAC increases myocardial protein expression of BDH1, which is associated with increased ketone body oxidation as indicated by increased ^13^C enrichment of acetyl CoA from ^13^C-β-hydroxybutyrate (Aubert et al., 2016). The shift toward greater ketone body utilization in compensated hypertrophy and/or heart failure has been postulated to be an adaptive response to maintain oxidative metabolism in the setting of decreased fatty acid oxidation. In further support that this metabolic alteration is adaptive, mice with a cardiac-specific deficiency of SCOT exhibit blunted myocardial ketone body oxidation, which is associated with accelerated pathological ventricular remodeling and cardiac dysfunction in response to TAC-induced pressure overload (Schugar et al., 2014). However, given that cardiac-specific SCOT-deficient mouse hearts also exhibited an increase in fatty acid oxidation, it remains unclear whether the increased pathological remodeling was attributed to the increase in fatty acid oxidation or the decrease in ketone body oxidation. Furthermore, it remains to be determined whether ketone body oxidation is increased in HCM that is characterized solely by diastolic dysfunction.

Taken together, a plethora of evidence has now been documented demonstrating that cardiac hypertrophy and heart failure are characterized by pronounced metabolic alterations. Whether these perturbations in myocardial energy metabolism contribute to the pathology of HCM, or whether they are due to the cardiac hypertrophy and subsequent dysfunction itself has not been conclusively determined. As mentioned previously, various intermediates of energy metabolism can directly regulate intracellular signaling, and one must also consider whether perturbations in myocardial energy metabolism regulate cardiac hypertrophy via such signaling changes. Indeed, the accumulation of glucose-6-phosphate as a result of elevated glycolysis in cardiac hypertrophy may directly promote ventricular hypertrophy, via activation of the mammalian target of rapamycin, a key signaling mediator of cardiac myocyte protein synthesis and subsequent growth (Shioi et al., 2003; Sen et al., 2013). Despite amino acids contributing minimally to myocardial energy metabolism, it has also been demonstrated that glucose can contribute to cardiac hypertrophy by preventing Kruppel-like factor 15 regulated transcription, thereby reducing branched chain amino acid metabolism and increasing mammalian target of rapamycin activation (Shao et al., 2018). Importantly, evidence is also limited that these metabolic alterations resulting from AAC- or TAC-induced cardiac hypertrophy are present and/or contribute to the pathology of genetic/familial HCM involving mutations in sarcomeric proteins. Nonetheless, HCM due to the AMPK γ2 subunit R302Q mutation does support that this form of familial HCM shares consistency with some of the documented changes in energy metabolism in cardiac hypertrophy.

## Energy Metabolism in Diabetic Cardiomyopathy

First described in diabetic patients with heart failure in the absence of myocardial ischemia, diabetic cardiomyopathy is characterized by ventricular dysfunction in the absence of underlying coronary artery disease and/or hypertension (Rubler et al., 1972). In addition, structural changes to the myocardium are present in diabetic cardiomyopathy, which include increased fibrosis and LV mass, and are often accompanied by underlying diastolic dysfunction with negligible impact on systolic function. Our understanding of diabetic cardiomyopathy has greatly improved since its original description in 1972, with numerous advancements regarding the mediators of its pathology. Some of the most extensively studied mechanisms include lipotoxicity, glucotoxicity, insulin resistance, alterations in calcium handling/signaling, fibrosis, endoplasmic reticulum stress, oxidative stress, cardiac myocyte apoptosis, and derangements in energy metabolism (Rubler et al., 1972; van de Weijer et al., 2011; Liu et al., 2012; Battiprolu et al., 2013; Ussher, 2014). With regards to the latter, myocardial energy metabolism in diabetic cardiomyopathy is substantially influenced by increased fatty acid delivery and insulin resistance, which leads to increases and decreases in fatty acid and glucose utilization, respectively. At a molecular level, increases in cardiac myocyte lipoprotein lipase activity lead to increased fatty acid delivery (Pulinilkunnil and Rodrigues, 2006; Kim et al., 2008), whereas hyperinsulinemia-mediated increases in cardiac myocyte protein kinase Cζ activity increase sarcolemmal CD36 protein expression and subsequent fatty acid uptake (Ouwens et al., 2007; Luiken et al., 2009). As fatty acid supply in obesity/type 2 diabetes (T2D) often exceeds increases in myocardial fatty acid oxidation rates, the build-up of myocardial lipid stores (TAG) and lipid intermediates (e.g., ceramide and diacylglycerol) frequently characterizes the diabetic myocardium (Lopaschuk et al., 2007; Zlobine et al., 2016). While the accumulation of TAGs, ceramides, diacylglycerols, and other lipid intermediates may promote myocardial insulin resistance, lipotoxicity, and cardiac myocyte apoptosis in diabetic cardiomyopathy, we encourage the reader to refer to the many excellent reviews already published on this topic (van de Weijer et al., 2011; D’Souza et al., 2016; Zlobine et al., 2016). The following section will detail the perturbations in myocardial energy metabolism that characterize the hearts of individuals with T2D (summarized in Table 2), and how these perturbations may potentially contribute to the pathology of diabetic cardiomyopathy.

**TABLE 2:** Alterations in myocardial energy metabolism in diabetic cardiomyopathy.

	Experimental model	Alteration in myocardial metabolism	References
Fatty acid metabolism	Male Obese Zucker rats	Fatty acid oxidation↓	Young et al., 2002
	Male *db*/*db* and *ob*/*ob* mice	Fatty acid oxidation↑	Mazumder et al., 2004; Buchanan et al., 2005; Hafstad et al., 2006; How et al., 2006
	Male C57BL/6J mice fed a high-fat diet	Fatty acid oxidation↑	Wright et al., 2009; Zhang et al., 2011
	Obese women, men and women with T1D, or men with T2D and non-ischemic cardiomyopathy	Fatty acid uptake↑ Fatty acid oxidation↑	Peterson et al., 2004; Herrero et al., 2006; Rijzewijk et al., 2009
Glucose metabolism	Male *db*/*db* and *ob*/*ob* mice	Glucose uptake↓ Glucose oxidation↓	Mazumder et al., 2004; Buchanan et al., 2005; Hafstad et al., 2006; How et al., 2006
	Male Wistar rats fed a high-fat and high-fructose diet, or male C57BL/6J mice fed a high-fat diet	Glucose uptake↓	Menard et al., 2010; Battiprolu et al., 2012
	Male C57BL/6J mice fed a high-fat diet	Glucose oxidation↓	Ussher et al., 2009; Wright et al., 2009
	Male Wistar rats or male C57BL/6J mice subjected to experimental T2D (high-fat diet + low-dose streptozotocin	Glucose oxidation↓	Le Page et al., 2015; Almutairi et al., 2020
Ketone body metabolism	Men and women with T2D undergoing cardiac catheterization	Ketone body uptake↑	Mizuno et al., 2017
	Male non-obese diabetic Goto-Kakizaki rats	Ketone body oxidation↑	Abdurrachim et al., 2019

While studies in isolated working hearts from fasted obese Zucker rats have shown that fatty acid oxidation rates are impaired (Young et al., 2002), the vast majority of literature has demonstrated that obesity and/or T2D lead to marked increases in myocardial fatty acid oxidation. For example, studies in genetic models of obesity/T2D have demonstrated that fatty acid oxidation rates are increased during aerobic perfusion of isolated working hearts from either 4 or 15-week-old male *db*/*db* and *ob*/*ob* mice (Buchanan et al., 2005). Similarly, fatty acid oxidation rates are increased during aerobic isolated working heart perfusions from male C57BL/6J mice after 2 weeks of high-fat feeding (45% kcal from lard) or 3 weeks of high-fat feeding (60% kcal from lard) (Wright et al., 2009; Zhang et al., 2011). These observations also translate to models of type 1 diabetes (T1D), as aerobic isolated working heart perfusions from rodents subjected to streptozotocin mediated destruction of islet β-cells, and Akita mice harboring a genetic mutation in the insulin 2 gene, demonstrate significant increases in fatty acid oxidation rates (Sakamoto et al., 2000; How et al., 2006; Basu et al., 2009). These observations have been recapitulated in humans, as positron emission tomography (PET) imaging with [1-^11^C]palmitate in obese women without cardiovascular disease, T1D subjects, or T2D subjects with non-ischemic cardiomyopathy, demonstrated significant increases in both myocardial fatty acid uptake and subsequent oxidation (Peterson et al., 2004; Herrero et al., 2006; Rijzewijk et al., 2009). It should be noted that many of these studies do not account for endogenous fatty acid oxidation from intracellular TAG stores, making it likely that fatty acid oxidation rates in preclinical and clinical studies of obesity/diabetes are actually being underestimated. As mentioned previously, myocardial TAG stores are increased in obesity/T2D. Furthermore, PET imaging studies with [1-^11^C]palmitate and [^1^H] magnetic resonance spectroscopy in obese patients without cardiovascular disease, reported that endogenous fatty acid oxidation rates are greater than exogenous plasma fatty acid oxidation rates, though a lean, healthy group for comparison was not included (Bucci et al., 2012). As such, these observations suggest that obesity/T2D increases the heart’s reliance on fatty acids from all sources (exogenous and endogenous) for energy metabolism. At a molecular level, PPARα is thought to be a key mediator of these metabolic alterations, as both T1D and T2D lead to increases in myocardial PPARα expression, and cardiac-specific PPARα overexpressing mice exhibit a cardiac phenotype mimicking that seen in diabetic cardiomyopathy (Finck et al., 2002, 2003). PPARα may also potentially be a key mediator of the increased reliance on TAG-derived fatty acid oxidation in obesity/T2D, as ^13^C nuclear magnetic resonance spectroscopy studies revealed increased TAG turnover in cardiac-specific PPARα overexpressing male mice fed a high-fat diet for 2 weeks (Banke et al., 2010). PPARα also contributes to the elevations in myocardial fatty acid uptake and subsequent lipotoxicity observed in obesity/T2D, which may be dependent on increased glycogen synthase kinase 3α (GSK3α) activity. It was demonstrated in neonatal rat cardiac myocytes that GSK3α can translocate to the nucleus and phosphorylate PPARα at serine residue 280, stimulating a biased PPARα transcriptional response that prompts increases in myocardial fatty acid uptake and lipid storage (Nakamura et al., 2019). Furthermore, mice with a cardiac-specific heterozygous GSK3α deficiency subjected to high-fat diet-induced obesity demonstrated significant improvements in diastolic function, as seen by a lower deceleration time during Tissue Doppler analysis. Conversely, increased myocardial PPARβ/δ may be protective against obesity/T2D-related cardiomyopathy, as cardiac-specific overexpression of PPARβ/δ prevents cardiac dysfunction following 8 weeks of high-fat diet supplementation (Burkart et al., 2007). This cardioprotection was attributed to PPARβ/δ increasing myocardial fatty acid oxidation but not fatty acid uptake, differing from PPARα activity, which increases both in the myocardium. Likewise, cardiac-specific deletion of PPARβ/δ in mice leads to a robust reduction in myocardial fatty acid oxidation, which results in a lipotoxic cardiomyopathy that leads to early mortality due to heart failure (Cheng et al., 2004).

With regards to impaired myocardial glucose utilization in obesity/T2D, cardiac myocytes isolated from adult *ob*/*ob* and *db*/*db* mice exhibit reduced insulin-stimulated Akt phosphorylation and 2-deoxyglucose uptake (Mazumder et al., 2004; Hafstad et al., 2006). Moreover, PET imaging with [^18^F]fluorodeoxyglucose demonstrated marked reductions in myocardial glucose uptake during a euglycemic-hyperinsulinemic clamp in male Wistar rats fed a high-fat and high-fructose diet for 6 weeks (Menard et al., 2010). Similarly, PET imaging revealed decreased [^18^F]fluorodeoxyglucose uptake in 12 h fasted male C57BL/6J mice fed a high-fat diet for at least 25 weeks (Battiprolu et al., 2012). On the contrary, other studies have suggested that myocardial insulin sensitivity remains intact in T2D, especially in the context of hyperinsulinemia and when plasma free fatty acid levels are matched (Lopaschuk et al., 2010). For example, 3 months of high-fat and high-cholesterol diet supplementation in male low-density lipoprotein receptor-deficient mice results in hyperinsulinemia and increased [^18^F]fluorodeoxyglucose uptake after a 4 h fast compared to standard chow fed lean mice (Gupte et al., 2013). Despite these divergent findings regarding myocardial glucose uptake in T2D, the marked impairment of myocardial glucose oxidation in the diabetic heart has been demonstrated in numerous studies. Aerobic perfusion of isolated working hearts from *ob*/*ob* mice, *db*/*db* mice, mice subjected to experimental obesity via high-fat diet supplementation, or mice and rats subjected to experimental T2D via high-fat diet supplementation plus low-dose streptozotocin administration, demonstrate impaired basal and insulin-stimulated glucose oxidation rates (Buchanan et al., 2005; Hafstad et al., 2006; How et al., 2006; Ussher et al., 2009; Wright et al., 2009; Le Page et al., 2015). The impaired glucose oxidation in the heart in the setting of obesity/T2D is partially attributed to the previously discussed “Randle Cycle” phenomenon, whereby elevated myocardial fatty acid oxidation rates decrease glucose oxidation via substrate competition (Lopaschuk et al., 2010). Reductions in MCU activity may also be involved, as C57BL/6NHsd mice subjected to experimental T1D via 5 daily injections with streptozotocin (40 mg/kg) exhibit decreased myocardial MCU protein expression and mitochondrial calcium levels in intact-paced contracting cardiac myocytes (Suarez et al., 2018). These changes are associated with reduced myocardial PDH activity and glucose oxidation rates during isolated working heart perfusions, but are completely normalized in mice with T1D administered an adeno-associated virus expressing murine MCU mRNA via the jugular vein. In addition, molecular control once again may involve increased PPARα activity, as PDHK4 mRNA expression, a transcriptional target of PPARα and the most prominent PDHK isoform in the heart, is increased in cardiac-specific PPARα overexpressing mice (Wu et al., 2001; Finck et al., 2002). On the contrary, in the setting of obesity/T2D, increased myocardial forkhead box O1 (FoxO1) may contribute to increased PDHK4-mediated inhibition of PDH activity and subsequent glucose oxidation. Indeed, PDHK4 is also a transcriptional target of FoxO1, and pharmacological FoxO1 antagonism with the agent AS1842856 increases glucose oxidation rates in aerobically perfused isolated working hearts from male C57BL/6J mice (Gopal et al., 2017). Furthermore, cardiac-specific FoxO1 deficiency prevents chronic high-fat diet supplementation mediated increases in myocardial PDHK4 mRNA expression (Battiprolu et al., 2012). The marked reduction in myocardial glucose oxidation is thought to directly contribute to the pathology of diabetic cardiomyopathy via decreasing cardiac efficiency, whereby ATP utilization for non-contractile purposes (e.g., ionic homeostasis) is increased (Lopaschuk et al., 2007; Almutairi et al., 2020).

Although our knowledge of myocardial ketone body oxidation has grown considerably with regards to its perturbations in cardiac hypertrophy and heart failure, this is an area of limited study in the diabetic myocardium. Nonetheless, it is imperative we increase our understanding of myocardial ketone body oxidation, as it has been suggested that the marked improvement in cardiovascular outcomes in people with T2D taking sodium-glucose cotransporter 2 (SGLT2) inhibitors (e.g., empagliflozin) may be due to increased ketosis and subsequent ketone body utilization (Ferrannini et al., 2016; Lopaschuk and Verma, 2016). Studies to date suggest that ketone body utilization may be increased in T2D, as catheterization studies for blood sampling from the coronary sinus and aortic root in subjects with T2D and mild diastolic dysfunction revealed increased myocardial uptake of acetoacetate and β-hydroxybutyrate (Mizuno et al., 2017). Furthermore, a recent study in non-obese diabetic male Goto-Kakizaki rats using hyperpolarized [3-^13^C]acetoacetate demonstrated increased myocardial ketone body utilization, as well as increases in myocardial SCOT activity (Abdurrachim et al., 2019). However, as these rats also demonstrated cardiac hypertrophy and systolic dysfunction, whether their metabolic perturbations are due to diabetes or systolic dysfunction cannot be discerned. Of interest, the increase in myocardial SCOT activity observed in diabetic male Goto-Kakizaki rats is also seen in skeletal muscles from C57BL/6J mice after 10 weeks of high-fat diet (60% kcal from lard) supplementation (Al Batran et al., 2020), suggesting that obesity/T2D may cause an increase in cardiac and skeletal muscle ketone body oxidation. While increased myocardial ketone body oxidation is a postulated mechanism by which SGLT2 inhibitors improve cardiovascular outcomes in T2D, careful consideration should be taken with regards to systemically boosting ketone body oxidation in humans with T2D, as increases in skeletal muscle ketone body oxidation appear to promote hyperglycemia (Al Batran et al., 2020).

## Energy Metabolism in Barth Syndrome

Despite a decreased prevalence with respect to the adult population, pediatric cardiomyopathies and heart failure are associated with a significant mortality rate and cost of care burden (Woulfe and Bruns, 2019). Many inherited cardiomyopathies affecting children involve inborn errors of metabolism that cause derangements in myocardial fatty acid oxidation, or mutations in mitochondrial DNA that impair normal oxidative phosphorylation, both of which have been reviewed extensively (Kelly and Strauss, 1994; Lee et al., 2017; Towbin and Jefferies, 2017). In this particular section, we will focus on pediatric cardiomyopathy associated with the rare genetic disorder, BTHS, which is caused by mutations in the *TAZ* gene, encoding for tafazzin, a mitochondrial transacylase critical for catalyzing the final step in cardiolipin remodeling (Jefferies, 2013; Dudek and Maack, 2017). Cardiolipin is a structurally distinct mitochondrial phospholipid with essential roles in the maintenance of mitochondrial morphology, regulation of mitochondrial protein transport and dynamics, and in maintaining the integrity and optimal activity of the ETC (Dudek and Maack, 2017; Dudek et al., 2019). Furthermore, aberrant myocardial cardiolipin content and composition, specifically reduction of the predominant tetralinoleoyl cardiolipin species, has been reported in a variety of cardiac pathologies, including idiopathic dilated cardiomyopathy (Chatfield et al., 2014), and in both human and experimental models of heart failure (Sparagna et al., 2007). As such, infantile-onset cardiomyopathy is the most common clinical feature associated with BTHS, although other phenotypic traits can include neutropenia, skeletal myopathy, exercise intolerance, 3-methylglutaconic aciduria, and pre-pubertal growth retardation (Jefferies, 2013). The cardiomyopathy associated with BTHS is primarily dilated cardiomyopathy, though HCM has also been documented, and may co-present with LV non-compaction or endocardial fibroelastosis (Spencer et al., 2006; Jefferies, 2013). In addition, the lack of a genotype-phenotype correlation suggests that physiological modifiers may exacerbate the tafazzin deficiency and contribute to the variability in clinical phenotypes of BTHS. However, due to cardiolipin’s key role in maintaining the ETC, perturbations in myocardial energy metabolism may also contribute to BTHS associated cardiomyopathies, which will now be highlighted in the following section (summarized in Table 3).

**TABLE 3:** Alterations in myocardial energy metabolism in BTHS.

	Experimental model	Alteration in myocardial metabolism	References
Fatty acid metabolism	Isolated cardiac mitochondria from male TazKD mice	Fatty acid oxidation↓ Fatty acid oxidation enzyme – ETC supercomplex interaction↓	Kiebish et al., 2013; Huang et al., 2015
	Neonatal cardiac myocytes from TazKD mice	Fatty acid oxidation↓	Powers et al., 2013
	Young adult males with BTHS	Fatty acid extraction↓	Cade et al., 2019
Glucose metabolism	Young adult males with BTHS	Glucose uptake↑ Glucose utilization↑	Cade et al., 2019
	Neonatal cardiac myocytes from TazKD mice	Glycolysis↑	Powers et al., 2013
Ketone body metabolism	Males (age range 6 months–32 years) with BTHS	Circulating β-hydroxybutyrate levels↑	Sandlers et al., 2016

*In vitro* studies have demonstrated that siRNA mediated *Taz* knockdown in neonatal rat ventricular cardiac myocytes decreases ATP levels, which was associated with increases in AMPK activity, cell surface area, and increased mRNA expression of brain natriuretic peptide, a biomarker of cardiac hypertrophy/heart failure (He, 2010). Decreases in ATP content are consistent with an impairment of oxidative phosphorylation, and cardiac mitochondria isolated from a mouse model of BTHS due to doxycycline mediated *Taz* knockdown (herein referred to as TazKD mice), display markedly reduced respiration, primarily attributed to impaired complex III activity (Kiebish et al., 2013; Powers et al., 2013). In young adults with BTHS (mean age of 26 years), a decreased myocardial energy reserve has also been observed, as indicated by a reduced phosphocreatine/ATP ratio during ^31^P magnetic resonance spectroscopy studies (Cade et al., 2019).

Despite well-characterized myocardial energy deficiency in BTHS, the actual alterations in myocardial intermediary energy metabolism are still being delineated. Paralleling the setting of heart failure, preclinical and clinical studies suggest that BTHS is associated with reductions in myocardial fatty acid oxidation. Isolated cardiac mitochondria from 3-month-old male TazKD mice subjected to proteomics analysis demonstrated disruption of ETC supercomplexes with the fatty acid oxidation enzymes, very long-chain acyl CoA dehydrogenase and long-chain 3-hydroxyacyl CoA dehydrogenase (Huang et al., 2015). Likewise, cardiac mitochondria isolated from 2-month-old male TazKD mice demonstrated a 25% reduction in palmitoyl-L-carnitine supported fatty acid oxidation during state 3 respiration, as determined using high-resolution respirometry (Kiebish et al., 2013). Oxygen consumption rates using a Seahorse XF24 analyzer were also reduced in neonatal cardiac myocytes isolated from TazKD mice, once more suggesting that BTHS is associated with impaired myocardial fatty acid oxidation (Powers et al., 2013). Illustrating the translatability of these findings, young adults with BTHS (mean age of 26 years) exhibited reductions in myocardial fatty acid extraction as determined by PET imaging (Cade et al., 2019).

With regards to BTHS associated changes in myocardial glucose metabolism, tafazzin deficiency mediated reductions in tetralinoleoyl cardiolipin formation may influence glucose metabolism through the regulation of PDH activity. Studies in C2C12 myoblasts subjected to CRISPR/Cas9 mediated *Taz* knockout demonstrated decreased incorporation of [U-^13^C]glucose into acetyl CoA, which was associated with an ∼50% inhibition of PDH activity (Li et al., 2019). Of interest, incubation of *Taz* knockout C2C12 myoblast mitochondria with exogenous cardiolipin restored this defect, providing mechanistic support for cardiolipin having a direct regulatory role on PDH activity. While C2C12 myoblasts originate from mouse skeletal muscle, high-resolution respirometry in cardiac mitochondria isolated from 2-month-old TazKD mice revealed no impairment in pyruvate supported state 3 respiration (Kiebish et al., 2013). Likewise, significant increases in myocardial glucose extraction fraction, uptake, and utilization were observed in young adults with BTHS (mean age of 26 years) compared to healthy age-matched controls (Cade et al., 2019). While these contrasting findings in humans suggest that BTHS may be associated with increased myocardial glucose oxidation, it is important to note that glucose oxidation was not directly assessed in the aforementioned study. It remains possible that the increase in myocardial glucose uptake and extraction reflects increases in myocardial glycolysis rates that are not coupled to a proportional increase in glucose oxidation. Evidence supporting this paradigm has been observed in neonatal cardiac myocytes isolated from TazKD mice, whereby an increased rate of extracellular acidification (indicative of increased glycolysis) was reported using a Seahorse XF24 analyzer (Powers et al., 2013). Furthermore, circulating lactate levels were increased in ∼4–5-month-old male TazKD mice in response to exercise, supporting that the increase in glucose utilization was not matched by a corresponding increase in glucose oxidation (Powers et al., 2013). Similar to what we have described in HCM and diabetic cardiomyopathy, the uncoupling of glucose oxidation from glycolysis may precipitate contractile inefficiency and contribute to the pathology of BTHS-related cardiomyopathy, as ATP is diverted toward supporting non-contractile purposes.

Regarding ketone metabolism, there has been limited investigation regarding potential alterations in myocardial ketone body utilization in BTHS. However, metabolomics in plasma collected from 23 subjects with BTHS demonstrated increases in circulating β-hydroxybutyrate levels compared to plasma from 15 age-matched control subjects not known to have an inborn error of metabolism (Sandlers et al., 2016). These findings are compatible with other preclinical and clinical studies that have noted a metabolic shift toward increased myocardial ketone body utilization in both cardiac hypertrophy and heart failure (Aubert et al., 2016; Bedi Jr., Snyder et al., 2016; Ho et al., 2019).

Although the metabolic profile characterizing the heart in BTHS remains the subject of ongoing study, it is evident that perturbations in intermediary energy metabolism are present, and it will be important to confirm whether these metabolic perturbations contribute to the pathology of BTHS-related cardiomyopathy. As a rare genetic disease, human BTHS studies of myocardial metabolism are limited in nature, and the furthering of our understanding of energy metabolism as a pathological mediator in this disorder will likely come from studies in the TazKD murine mouse model of BTHS (Acehan et al., 2011). However, cardiac phenotypes reported in TazKD mice are not consistent in the literature, with some studies demonstrating the more prevalent dilated cardiomyopathy seen in BTHS subjects (Acehan et al., 2011), whereas others have reported a HCM that is less frequently observed in BTHS subjects (Johnson et al., 2018). As such, it will be important for future studies to carefully dissect the specific cardiac phenotypes present in TazKD mice, and how they may relate to potential contrasting findings relating to perturbed myocardial energy metabolism.

## Energy Metabolism in Arrhythmogenic Cardiomyopathy

Arrhythmogenic cardiomyopathy is another form of inherited cardiomyopathy that is genetically heterogenous, typically caused by genetic mutations producing abnormalities in cardiac desmosomes, and it is a major risk factor for sudden cardiac death. The reader is encouraged to refer to (Corrado et al., 2017) for extensive review of the various mutations that can predispose to arrhythmogenic cardiomyopathy. Depending on geographic region the overall prevalence of arrhythmogenic cardiomyopathy can range from 1:1000 to 1:5000, though most specialists in this area are of the opinion that the real prevalence is closer to the latter (Corrado et al., 2017). Although originally referred to as arrhythmogenic right ventricular dysplasia or arrhythmogenic right ventricular cardiomyopathy, increasing reports characterized by early and greater LV involvement have resulted in use of the broader arrhythmogenic cardiomyopathy term (Norman et al., 2005). Arrhythmogenic cardiomyopathy is characterized by ventricular arrhythmias, systolic dysfunction, and replacement of myocardium with fibrofatty tissue (Corrado et al., 2017). Few studies to date have performed any meaningful assessment of myocardial energy metabolism in arrhythmogenic cardiomyopathy and other heart rhythm disorders such as atrial fibrillation or ventricular tachycardia, though there are reports alluding to perturbations in energy metabolism.

In a rat model of stress-induced cardiac damage and arrhythmias, administration of an overdose of isoproterenol (67 mg/kg) to male Wistar rats produces a mild systolic dysfunction and notable diastolic dysfunction in a hypercontractile state 2 weeks following isoproterenol treatment (Willis et al., 2015). Moreover, isolated mitochondria from the hearts of these rats at the same time point demonstrated significant reductions in both NADH and succinate linked respiration, whereas monitoring of aconitase activity demonstrated increases in oxidative stress. In 12 to 16-week old male B6.129S mice fed a high saturated fat diet (60% kcal from palm oil) for 4 weeks that does not induce significant weight gain (∼3–4 grams) or cardiac dysfunction, *in vivo* telemetry revealed ventricular ectopy and a prolonged QT period due to an increase in NADPH oxidase 2 mediated oxidative stress (Joseph et al., 2019). Of interest, these observations were absent in mice with a whole-body deficiency of NADPH oxidase 2. Metabolic perturbations may also contribute to atrial fibrillation, as atrial expression of fatty acid binding protein 3, a key regulator of cellular fatty acid uptake and subsequent transport, is elevated in right atrial tissue biopsies from Japanese individuals undergoing heart surgery with established atrial fibrillation versus those with normal sinus rhythm (Shingu et al., 2020). Last, the cardiac ryanodine receptor (RYR2) plays a key role in excitation-contraction coupling, whereby sarcoplasmic reticulum release of calcium through the RYR2 allows for calcium to interact with cytosolic contractile proteins, and both gain and loss of function RYR2 mutations can promote arrhythmias and sudden cardiac death (Gomez and Richard, 2004). Studies in mice with a 50% reduction in cardiac RYR2 protein expression demonstrate significant reductions in heart rate assessed via *in vivo* radiotelemetry, which is associated with reductions in glucose oxidation rates assessed via isolated working heart perfusions (Bround et al., 2016).

While the above described studies lend credence to the notion that perturbations in energy metabolism may contribute to heart rhythm disturbances that are often present in people with arrhythmogenic cardiomyopathy, it should be noted that none of these studies actually assessed metabolic flux *per se*. Furthermore, whether these metabolic perturbations are causally related to heart rhythm disturbances or simply the result of arrhythmias remains to be determined, and it is also currently unknown whether such observations would translate to the genetic mutations that predispose to inherited arrhythmogenic cardiomyopathy.

## Energy Metabolism as a Target to Alleviate Cardiomyopathy

As previously discussed, numerous studies have demonstrated that during the transition from compensated hypertrophy to decompensated heart failure, myocardial fatty acid oxidation rates decline as the heart adopts a phenotype mimicking fetal metabolism. Hence, the preservation of fatty acid oxidation has been investigated as a potential therapeutic strategy to reverse maladaptive metabolic remodeling and preserve cardiac function in the setting of pathological HCM. Cardiac-specific deletion of acetyl CoA carboxylase 2 in mice, which increases myocardial fatty acid oxidation via preventing the synthesis of malonyl CoA, is associated with reductions in cardiac hypertrophy and fibrosis, as well as improved *ex vivo* and *in vivo* cardiac function at 8 weeks post-TAC (Kolwicz Jr., Olson et al., 2012). However, as a genetic model where myocardial fatty acid oxidation rates are chronically elevated, it remains uncertain whether stimulating myocardial fatty acid oxidation specifically at the compensated hypertrophy stage, and prior to systolic dysfunction, would yield a similar result. Although there is inconsistency regarding myocardial glucose oxidation alterations in cardiac hypertrophy, treatment of isolated working hearts from male Sprague-Dawley rats after 10 weeks of AAC with dichloroacetate (DCA, 2 mM), a PDHK inhibitor, improves *ex vivo* cardiac function as indicated by increased LV pressures (Pound et al., 2009). In addition, infusion of angiotensin II (1.5 mg⋅kg^–1^⋅day^–1^), a potent vasoconstrictor and mediator of cardiac injury, for 14 days via implanted micro osmotic pumps in male C57BL/6J mice induces cardiac hypertrophy and diastolic dysfunction (Mori et al., 2012). Moreover, isolated working hearts from these mice demonstrated significant reductions in glucose oxidation rates during aerobic perfusion, concomitant with a reduction in PDH activity. Interestingly, treatment with irbesartan (50 mg⋅kg^–1^⋅day^–1^), an angiotensin II type 1 receptor antagonist, prevents angiotensin II infusion-induced cardiac hypertrophy and diastolic dysfunction, which is associated with increased myocardial PDH activity and subsequent glucose oxidation rates. It should be noted, though, that it remains unclear whether the increase in myocardial glucose oxidation rates is mechanistically required for how irbesartan attenuates diastolic dysfunction in response to angiotensin II infusion. Myocardial anaplerosis can also be targeted to attenuate cardiac dysfunction in response to TAC-induced pressure overload in male Sprague Dawley rats, as adenoviral delivery of a microRNA specific to malic enzyme improved contractile function during isolated Langendorff perfusions at 12 weeks post-TAC (Lahey et al., 2018). Taken together, it does appear that modifying myocardial energy metabolism can mitigate cardiac dysfunction in experimental models of cardiac hypertrophy. Nevertheless, due to inconsistencies in the models utilized, as well as whether systolic or diastolic dysfunction predominates, or whether both are present, makes it challenging to discern whether targeting myocardial energy metabolism can mitigate the diastolic dysfunction often observed in genetic/inherited HCM.

With regards to diabetic cardiomyopathy, numerous preclinical studies have demonstrated that correcting either elevated myocardial fatty acid oxidation rates or impaired myocardial glucose oxidation rates, yields salutary actions on cardiac function. For example, treatment for 3 weeks with the antianginal agent, trimetazidine (15 mg/kg once daily), which decreases fatty acid oxidation by inhibiting 3-ketoacyl-CoA thiolase, prevented diastolic dysfunction in 26-week-old male C57BL/6J mice fed a high-fat diet for 13 weeks (Ussher et al., 2014). These findings may translate to humans with T2D, as trimetazidine treatment for 6 months (20 mg 3x daily) in subjects with T2D and idiopathic dilated cardiomyopathy ameliorated both systolic and diastolic dysfunction (Zhao et al., 2013). Moreover, mice with a whole-body deficiency of malonyl CoA decarboxylase demonstrated reductions in fatty acid oxidation due to elevated malonyl CoA levels, while also displaying marked improvements in cardiac efficiency following high-fat diet-induced obesity (Ussher et al., 2009). As the reduction in myocardial fatty acid oxidation in response to treatment with trimetazidine, or due to malonyl CoA decarboxylase deficiency, is accompanied by corresponding increases in myocardial glucose oxidation, directly stimulating glucose oxidation in the heart also yields salutary actions in diabetic cardiomyopathy. In particular, supplementation of the drinking water for 4 weeks with DCA (1 mM), augmented myocardial PDH activity and glucose oxidation rates in male Wistar rats subjected to experimental T2D via high-fat diet supplementation and low-dose streptozotocin (25 mg/kg) administration (Le Page et al., 2015). Importantly, diastolic dysfunction was also ameliorated in these rats following treatment with DCA. Similarly, liraglutide, a T2D therapy that improves cardiovascular outcomes (Al Batran et al., 2018), also increases glucose oxidation rates in male C57BL/6J mice subjected to experimental T2D via high-fat diet supplementation and low-dose streptozotocin (75 mg/kg) administration, which was once again accompanied by an alleviation of diastolic dysfunction (Almutairi et al., 2020). On the contrary, increasing myocardial fatty acid oxidation via cardiac-specific deletion of acetyl CoA carboxylase 2 alleviates both systolic and diastolic dysfunction in a mouse model of obesity-induced cardiomyopathy following 24 weeks of supplementation with a high-fat diet (Shao et al., 2020). As mentioned previously, it has been suggested that SGLT2 inhibitors (e.g., empagliflozin) may improve cardiovascular outcomes in people with T2D via increasing myocardial ketone body oxidation rates (Lopaschuk and Verma, 2016). While cardiovascular outcomes studies with SGLT2 inhibitors have not assessed diabetic cardiomyopathy *per se*, preclinical studies have demonstrated that the SGLT2 inhibitor, empagliflozin, can attenuate diastolic dysfunction in obesity-induced cardiomyopathy (Sun et al., 2020). Nonetheless, whether myocardial ketone body oxidation rates were increased was not assessed, and treatment of genetically obese *db*/*db* mice with empagliflozin did not increase ketone body oxidation rates assessed during aerobic isolated working heart perfusions (Verma et al., 2018). Therefore, future studies are necessary to further elucidate whether modulating myocardial ketone body oxidation can meaningfully impact the pathology of diabetic cardiomyopathy, and whether this may represent a potential cardioprotective mechanism of SGLT2 inhibitors.

Although there are no curative therapies for BTHS, BTHS-related cardiomyopathy is primarily managed using standard heart failure pharmacotherapy. However, in light of the documented myocardial metabolic perturbations in BTHS previously discussed, pharmacological optimization of myocardial energetics may represent a promising therapeutic approach to attenuate this form of inherited cardiomyopathy. To date, few studies have examined whether correcting defects in myocardial energy metabolism can reduce the risk for cardiomyopathy in people with BTHS. Intriguingly, treatment of 3-month-old TazKD mice with the pan-PPAR agonist (primarily PPARα), bezafibrate, via supplementation in the diet (0.05% weight/weight) for 4 months, prevented the development of dilated cardiomyopathy and systolic dysfunction (Schafer et al., 2018). Despite myocardial energy metabolism not being directly assessed, gene-ontology analysis revealed that bezafibrate treatment resulted in increased expression of genes involved in multiple intermediary energy metabolism pathways, and the actions of PPAR agonists to increase fatty acid oxidation are well documented (Lopaschuk et al., 2010). Nonetheless, Aasum et al. (2008) have shown that systemic administration of fibrates decreases myocardial fatty oxidation rates due to an elevation of hepatic fatty acid oxidation, which lowers circulating free fatty acids and TAGs, thereby decreasing fatty acid delivery to the heart. This decrease precipitated a corresponding increase in myocardial glucose oxidation and improvement in cardiac efficiency, suggesting that the potential benefit seen with bezafibrate therapy in TazKD mice may involve a similar mechanism. Furthermore, a mitochondrial targeted antioxidant that binds selectively to and stabilizes cardiolipin, elamipretide, is being investigated as a novel therapy for heart failure (Birk et al., 2013). While studies in a swine model of obesity and *ex vivo* human failing heart samples have demonstrated that elamipretide can improve mitochondrial function and integrity, it would be of interest to assess its potential utility for correcting cardiolipin defects in BTHS, and whether that is associated with a normalization of myocardial energy metabolism. Overall, future studies are still required to further delineate the specific metabolic perturbations that characterize the myocardium in BTHS, in order to better assess whether therapeutic interventions aimed at modulating myocardial energy metabolism may indeed be beneficial.

## Final Summary

Both ischemic and non-ischemic dilated cardiomyopathies often lead to overt heart failure and are characterized by perturbations in myocardial energy metabolism that may represent modifiable targets for reversing disease pathology (Taha and Lopaschuk, 2007; Jaswal et al., 2011; Heusch et al., 2014; Gibb and Hill, 2018). As our understanding of other forms of cardiomyopathies (e.g., HCM, diabetic cardiomyopathy, BTHS-related cardiomyopathy, arrhythmogenic cardiomyopathy) continues to grow, it does appear that perturbations in myocardial energy metabolism may actually represent a shared feature of cardiomyopathy in general. Before optimization of myocardial energy metabolism should even be considered as a possible therapeutic approach to improve cardiac function in genetic/inherited HCM, it will be important to develop novel mouse models harboring the same genetic mutations responsible for the numerous inherited HCMs identified to date. Indeed, BTHS represents a form of genetic/inherited cardiomyopathy where substantial advancements in defining myocardial energy metabolism defects have been made, due to the generation of the TazKD mouse model. Likewise, as non-invasive *in vivo* imaging techniques improve for quantifying myocardial energy metabolism in humans (e.g., hyperpolarized ^13^C magnetic resonance imaging to assess glucose oxidation), future opportunities to advance our understanding of myocardial energy metabolism in genetic/inherited HCM will likely follow. These same issues also apply to and should be considered in arrhythmogenic cardiomyopathy. If both avenues of pursuit indicate metabolic perturbations that parallel those observed in cardiac hypertrophy or cardiac arrhythmias, then identifying novel ways to feasibly reverse these defects in myocardial energy metabolism with pharmacotherapies should be explored. Finally, the perturbations in myocardial energy metabolism characterizing diabetic cardiomyopathy have been extensively studied, and appear to be a promising approach for improving cardiac function in people with T2D. Therefore, as we continue to further understand the metabolic perturbations present in various cardiomyopathies, while delineating the molecular mechanisms responsible, it should pave the way for unique targets that can be modified using a personalized medicine approach to treat human cardiomyopathy.

## Author Contributions

All authors researched literature, drafted and wrote the review article, and approved the submitted version.

## Conflict of Interest

The authors declare that the research was conducted in the absence of any commercial or financial relationships that could be construed as a potential conflict of interest.
